# Cardiac Stimulation for Suspended Animation by Direct Digital Manipulation—Illustrated by Diagrams and Case Reports

**Published:** 1907-01

**Authors:** Benjamin Merrill Ricketts

**Affiliations:** Cincinnati, O.


### CARDIAC STIMULATION FOR SUSPENDED ANIMATION BY DIRECT DIGITAL MANIPULATION—ILLUSTRATED BY DIAGRAMS AND CASE REPORTS.

---

A Supplementary Report to Surgery of The Heart and Lungs.

By BENJAMIN MERRILL RICKETTS, M. D., LL. D., Cincinnati, O.

---

Read before the Surgical Section American Medical Association, Boston, Mass., June 5, 6, 7 and 8th, 1906.

The definition of life is: The functions depending directly on the cerebro-spinal nervous system and the voluntary muscles.

The definition of death: Cessation of the heart beat.

The various methods for determining death are considered, among them that of Foubert, who bases his sign of death upon this definition. He says, if the heart is found not to pulsate, dissolution has taken place. He introduces the finger through the intercostal space to the heart to determine this.

With him the introduction of the finger was to condemn to death. Now the introduction of the finger is to restore life.

No known methods of determining death are positive in non-multilated bodies within several minutes after cessation of the heart beat.

For this reason all known methods for resuscitation should be employed when the heart ceases to beat until a time when positive methods of death can be applied.

Cardiac contraction has been induced many hours and even days after death had been made positive.

Many lives have been unnecessarily sacrificed for the want of effort to save them.

No one method should be employed to the exclusion of all others until more definite knowledge upon this subject is btained by experimentation and observation upon the various forms of animals, both living and dead.

The case at Brighton, England, November 11, 1905, is cited as an illustration to show the force of this argument. The child nine days old lived sixteen hours after the physician had pronounced it to be dead.

The class of cases in which rythmic massage of the heart is appropriate is suspended animation due to either chloroform, nitrous oxide, carbon dioxide, shock due to fright, injury or surgical operation, drowning, electrocution, strangulation, loss of blood and probably disease, and drugs of many kinds.

Experimental resuscitation by rythmical cardiac compression was first practiced by Schiff in 1874 and Ricketts in 1877. Defibrinated blood, sodium, adrenalin, chloride and Lock's solution are mention as of especial interest.

Hearts removed from both the live and dead body have been subjected to heat and cold, both dry and moist, for periods of time varying in length, and found to contract when subjected to the electric current.

The experiments of Westbrook 1882, Prus 1889, Velich 1896, Porter 1897, Ricketts 1899, Batelli 1900, Kuliabke 1902, Spina 1903, Crile 1903, D'Halluin 1904, Kemp and Gardener 1904, Muller 1905, with others, have shown that contractions of the heart have been induced in many ways under many abnormal circumstances.

It has been shown that lack of potassium salts renders the vagus wholly or nearly ineffective and the increase of calcium has no effect on vagus control.

Intro-Theracic Transperi, cardial cardiac massage, was first done by Nichaus in 1880. It is done after making a horse-shoe incision through the cutaneous structure and dividing the third, fourth and fifth ribs from right to left or left to right preferably turning the flap from right to left . The hæmorrhage is insignificant unless the internal mammary artery is injured. The pericardium is exposed and divided that the heart may be grasped between the thumb and forefinger. Care should be taken not to open the pleural cavity, but if this has been done the opening of the pleura should be disregarded.

Of the twenty-one cases reported, two were complete recoveries, eleven lived from one minute to twenty-seven hours, and nine the pulse never returned. In nineteen the chest was deliberately opened, two the chest was already opened. Sixteen chloroform was used, and four not stated. In one recovery, the heart did not beat for three minutes, while in another it did not beat for one minute. Of those that died, in one the heart did not pulsate for sixty seconds, then lived for twenty-seven hours before dissolution. In two others the heart did not beat for fifteen minutes; one lived sixteen hours before final dissolution.

Sub-Diaphragmatic Transperitoneal Cardiac Massage is supposed to have been first done by Starling and Land in 1902. The heart is grasped with the hand with the diaphragm intervening. Eighteen have been treated in this way, eight of which the abdomen was deliberately opened for the purpose, and ten the abdomen had already been opened for other purposes; three chloroform was used, ten not stated. Three the diaphragm was deliberately opened from below to massage the heart. Ten permanently recovered; eight died, five of which the pulsations were restored for a short time, and three the pulsations were not restored. Of the eight in which the chest was deliberately opened, three recovered; and of the ten cases in which the abdomen had already been opened, six recovered. Of the ten recoveries, one the heart ceased to beat one minute, one four minutes, one ten minutes, one, one minute plus the time required to open the abdomen, and seven not stated.

The subject is presented in five chapters mainly.

I. Preamble.
II. Experimental.
III. Intra-theracic Trans-pericardial Massage.
IV. Sub-Diaphragmatic Trans-peritoneal Massage, and
V. Miscellaneous Bibliography.

Cases in which the heart beat was re-established:

Total cases operated, 39.

Primary opening of chest alone, 19; already open, 1.

Primary opening of abdomen alone, 8; already open, 9.

Opening of chest and abdomen, 2.

Number of chest operations recovered, 2—10 per cent.

Number of adbominal operations recovered, 10—62 1-2 per cent.

Number of diaphragms incised, 3; all died.

Eight abdomens deliberately opened; 3 recovered.

Nine abdomens already opened; 7 recovered.

Number of males, 16; females, 7; not stated, 16.

Number of chloroform administrations, 27; ether, 1; not stated, 11.

Number of cases in which the character of operation for which the anaesthetic was given, 25; not stated, 14.

Total number of cases in which the heart beat was r-established, 31.

Total number in which the heart beat was not re-established, 8.

Total number of cases in which the heart beat was permanently re-established, 12.

Number of ages given, 22.

Time before heart beat was re-established, one to sixty minutes.

Greatest length of time of cessation of heart beat to be re-established permanently was 20 minutes.

---

In dealing with infections of the hand bear in mind that under a simple bleb may lie an extensive phlegmon, threatening, or actually involving, a tendon or bone and urgently needing a generous but wisely placed incision; while on the other hand, a tendon may be thrust from its protecting sheath into the area of destruction by a knife sweep more earnest than judicious. A crater-like opening in a sodden skin though freely discharging pus, may need enlarging to protect the tissues underlying; while another opening, too long continued by unnecessary packing, may cripple a joint or tendon by undue cicatrization.—*American Journal of Surgery.*

---

Wet dressings, especially the evry useful Burow's solution of aluminum acetate, when applied to the hand or foot, usually cause maceration and whitening of the skin, which is apt to alarm the patient. The addition to the solution of one-fourth its bulk of glycerin or alcohol, will obviate this unsightly maceration.—*American Journal of Surgery.*

